# Nuclear Envelope Dynamics in *Dictyostelium* Amoebae

**DOI:** 10.3390/cells14030186

**Published:** 2025-01-26

**Authors:** Ralph Gräf, Petros Batsios, Marianne Grafe, Irene Meyer, Kristina Mitic

**Affiliations:** 1 Department of Cell Biology, University of Potsdam, Karl-Liebknecht-Str. 24-25, 14476 Potsdam-Golm, Germany; mgrafe@uni-potsdam.de (M.G.); irene.meyer@uni-potsdam.de (I.M.); mitic@uni-potsdam.de (K.M.); 2 Sigma-Aldrich Chemie GmbH, Eschenstraße 5, 82024 Taufkirchen, Germany; petros.batsios@merckgroup.com

**Keywords:** nuclear envelope, nuclear pore complex, nuclear lamina, lamin, HeH family, *Dictyostelium*, mitosis, centrosome

## Abstract

In the last decades, the study of many nuclear envelope components in *Dictyostelium* amoebae has revealed conserved mechanisms of nuclear envelope dynamics that root back unexpectedly deep into the eukaryotic tree of life. In this review, we describe the state of the art in nuclear envelope research in this organism starting from early work on nuclear pore complexes to characterization of the first true lamin in a non-metazoan organism and its associated nuclear envelope transmembrane proteins, such as the HeH-family protein Src1 and the LINC complex protein Sun1. We also describe the dynamic processes during semi-closed mitosis, including centrosome insertion into the nuclear envelope, and processes involved in the restoration of nuclear envelope permeability around mitotic exit and compare them to the situation in cells with open or fully closed mitosis.

## 1. Introduction

The nuclear envelope is characterized by an outer nuclear membrane, which is in continuation with the endoplasmic reticulum, and an inner nuclear membrane (INM). The outer and inner nuclear membranes are separated by the perinuclear space and are connected to each other at the nuclear pore complexes (NPCs), which are responsible for specific nuclear transport. Consequently, the perinuclear space is in continuation with the lumen of the endoplasmic reticulum. In animal cells and *Dictyostelium* amoebae, the INM is associated with the nuclear lamina consisting of a meshwork of type V intermediate filaments, the lamins, and the associated nuclear envelope transmembrane proteins (NETs) of the INM.

Usually, animal or fungal models have been used to study nuclear envelope organization. Thus, among animals, most work focuses on mammalian cell cultures, *Caenorhabditis elegans* worms, and *Drosophila* flies, and among fungi, on the two yeasts *Saccharomyces cerevisiae* and *Schizosaccharomyces pombe*, plus the filamentous fungus *Aspergillus nidulans*. However, with regard to the evolution of the eukaryotic tree of life, this approach does not really cover the entire range of organisms. Animals and fungi both belong to the clade of *Opisthokonta*. To further extend the spectrum, about 25 years ago, the hitherto best-established amoebozoan model, *Dictyostelium discoideum*, emerged not only as a subject for ultrastructural studies but also for molecular analyses of the nuclear envelope. Early work started with the characterization of interaptin, a nesprin-like actin-binding protein located at the outer nuclear membrane [1], and continued with early molecular and ultrastructural studies of the NPC [2,3,4]. Subsequently, studies on the *Dictyostelium* nuclear envelope continued with the characterization of Sun1, one component of the LINC complex (linker of the nucleus and the cytoskeleton) [5,6,7,8,9], the nuclear envelope-associated nucleolus [10], the identification of the first lamin in a non-metazoan cell [11], and cell cycle-dependent changes in nuclear envelope permeability during semi-closed mitosis [12].

**Figure 1:**
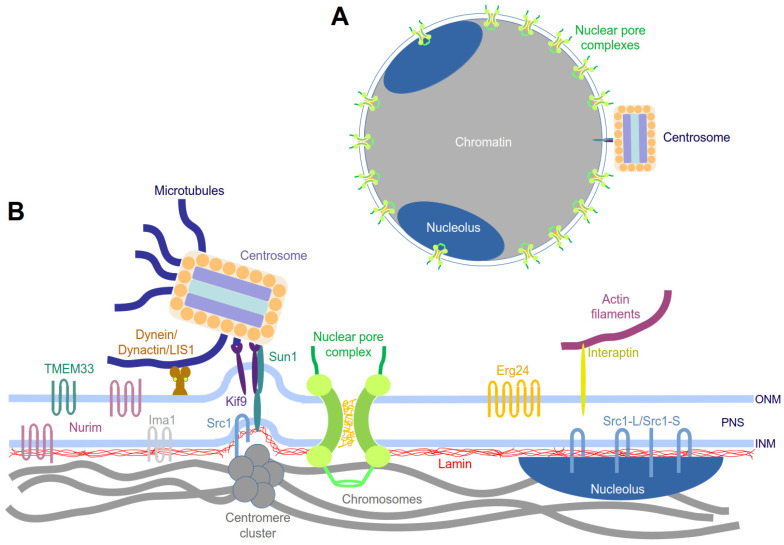
Structural organization of the nucleus (**A**) and nuclear envelope (**B**) of *Dictyostelium* amoebae. Characterized proteins are shown at their known localizations. Abbreviations: ONM, outer nuclear membrane; INM, inner nuclear membrane; PNS, perinuclear space. See main text for details.

Before we provide a detailed description of the current model of dynamic nuclear envelope organization and the known molecular players in *Dictyostelium* amoebae, we would like to give a general overview of nuclear envelope organization in this organism (Figure 1).

In vegetative (i.e., replicating) cells, the interphase nucleus has a diameter of approximately 2 µm and an almost perfectly spherical shape. It contains a haploid set of six subtelocentric chromosomes, whose centromeres are clustered in close vicinity to the nuclear envelope, always directly adjacent to the centrosome [13]. The centrosome, also called the nucleus-associated body (NAB), is attached to the cytosolic face of the nucleus. It contains no centrioles, and instead, it consists of a cylindrical core structure with three major layers, surrounded by a so-called corona harboring microtubule-nucleation complexes. The centrosome is bound to the nuclear envelope via a fibrous linkage, which connects it to the centromere cluster at the nuclear side (Figure 1B) [14]. The one to three nucleoli (most often there are two), easily visible in phase-contrast light microscopy, are attached to the nuclear envelope in opposition to the site of the centromere/centrosome (Figure 1A). At the G2/M transition, the nucleoli disappear and the centrosome enters a fenestra in the nuclear envelope and starts to duplicate [12,15,16]. The permeability of the nuclear membrane for spindle assembly factors and tubulin dimers increases, while the integrity of the nuclear membrane remains otherwise unchanged. Thus, *Dictyostelium* amoebae display a semi-closed mitosis, similar to fungi, such as *Aspergillus nidulans* [12,17,18].

Here, karyokinesis, i.e., the separation of the two daughter nuclei, proceeds with intact nuclear envelopes until the final abscission of the membranous connection between them [19]. Upon starvation, Dictyostelium amoebae employ a sophisticated cAMP-signaling process to gather via chemotactic streaming and to form a multicellular mound. Two cell types, stalk cells and spore cells, differentiate and form a fruiting body with a thin stalk and a spore head [20]. The spores survive unfavorable environmental conditions and hatch into amoebae again under favorable conditions. During the streaming process, approximately six hours after the beginning of starvation, the nuclei take on an oval shape whereby the nucleoli usually unite into one large nucleolus located at the opposite site of the nucleus with regard to the centrosome/centromere cluster [21].

## 2. The Nuclear Envelope

### 2.1. Lamins

As depicted in Figure 1, the nuclear lamina includes numerous direct or indirect interactions of the lamin network with various nuclear envelope-associated proteins and structures. Therefore, we use this structure as the starting point to discuss the nuclear envelope in more detail. Ultrastructural analyses undertaken already in the fifties of the last century revealed that the inner nuclear membrane is associated with a fibrous lamina [22,23,24,25], and already in the seventies, its first molecular components were characterized, which were called lamins [26,27]. Subsequently, these proteins were identified as members of the intermediate filament protein family. In mammals, three genes were identified encoding different lamin isoforms [28,29]. They were called *LMNA*, *LMNB1*, and *LMNB2*. *LMNA* encodes lamin A and its splice variant lamin C, while *LMNB1* encodes lamin B1 and *LMNB2* encodes lamin B2 and its splice variant lamin B3 in embryonal cells (also called lamin LIII in some species). Lamins are characterized by a typical intermediate filament-like domain pattern with an N-terminal head domain, followed by an α-helical rod domain of 370 amino acids and a C-terminal tail domain, including a nuclear localization sequence (NLS) and a conserved lamin tail domain (LTD). In addition, lamin A and all B-type lamins are characterized by a C-terminal CaaX-box (cysteine, two aliphatic amino acids, any amino acid), a signal for conjugation with an isoprenyl residue, which anchors the protein at membranes. The CaaX-box, and hence isoprenylation, is missing in lamin C due to alternative splicing of the lamin A pre-mRNA. Lamins are generally translated as pre-proteins, which are processed prior to their assembly into filaments [30]. The processing of pre-lamins by ER-bound enzymes starts with the binding of an isoprenyl residue (usually farnesyl or geranylgeranyl) to the cysteine side chain of the CaaX-box by farnesyl transferase. Processing continues with proteolytic cleavage of the three C-terminal amino acids by Ras-converting enzyme 1 (RCE1) or ZMPSTE24, followed by methylation of the now C-terminal isoprenylated cysteine by isoprenyl cysteine methyl transferase. The binding of importin α to the NLS of the nascent protein chain inhibits the assembly of filaments prior to nuclear import, after which the small GTPase Ran-GTP causes the dissociation of importin α to allow for filament assembly within the nuclear interior [31]. In the case of B-type lamins, the processing ends at this point, whereas in the case of lamin A, it continues with the cleavage of the last 15 amino acids by ZMPSTE24 only in the course of filament assembly. Superresolution light microscopy revealed that A-type lamins and B-type lamins form overlapping but independent filamentous networks formed by homo-oligomers beneath the nuclear membrane, whereby only B-type lamins are permanently anchored to the inner nuclear membrane via their isoprenyl anchor [32,33]. The latter also explains why B-type lamins remain associated with remnants of the former nuclear envelope even after nuclear envelope breakdown during mitosis [26] and why A-type lamins are also found in the nucleoplasm during interphase [34]. At least one B-type lamin is expressed in all mammalian cells, while A-type lamins are restricted mainly to cells in the course of differentiation, such as MEFs (mouse embryonal fibroblasts). This general presence of B-type lamins is in agreement with the fact that, in contrast to mammals, many other metazoans possess only one lamin gene, which belongs to the B-type [29]. This appears to be similar in non-metazoan organisms, which were initially thought to possess no lamins at all, although the presence of a nuclear lamina had been postulated based on early ultrastructural data (see above).

In *Dictyostelium*, already in the 1970s, a thin, continuous, low-electron-density layer was detected between the inner nuclear membrane and the chromatin [11], but it took until 2009, when in a search for new centrosome-associated proteins, a *Dictyostelium* protein was detected for which the amino acid sequence properties were reminiscent of a lamin [35]. It was called NE81, due to its calculated molecular mass and exclusive distribution along the nuclear envelope when expressed as a GFP-fusion protein. Its presence in isolated centrosomes could be explained by the co-purification of nuclear envelope fragments with centrosomes, caused by the tight association of the centrosome with the nuclear envelope (see below). In contrast to nuclear lamina proteins from higher plants and *Trypanosoma*, which are evolutionarily unrelated to lamins [36], the further molecular characterization of NE81 (see below) revealed that it was in fact the first true lamin of a non-metazoan organism [11,37,38]. Therefore, we now call it *Dictyostelium* lamin. The identification of this lamin and the advent of improved bioinformatic tools allowed for the subsequent identification of lamin-related protein sequences in organisms representing clades other than *Amoebozoa,* such as *Archaeplastida*, *Excavata*, and SAR (*Stramenopile, Alveolata, Rhizaria*) [39,40]. Meanwhile, it is widely accepted that lamins already belonged to the protein equipment of the last eukaryotic common ancestor (LECA) [36].

Only one lamin isoform could be identified in the genomes of various *Dictyostelium* species. Despite an only moderate level of overall conservation at the amino-acid-sequence level compared to animal lamins, the sequence pattern and the order of functional elements classifies NE81 as a lamin. It exhibits the typical pattern of a head domain, a 370-amino acid α-helical rod domain, and a tail domain with a nuclear localization sequence (NLS) at the N-terminal part, a lamin tail domain (LTD), and a CaaX-box at the C-terminal part. The head domain also includes a conserved consensus sequence for phosphorylation by cyclin-dependent kinase 1 (CDK1) at position 122. Our studies confirmed that the protein does not only appear like a lamin on the sequence level, but it also behaves like a lamin on the functional level.

Both the overexpression, as well as the knockout, of *NE81* [11] resulted in disorganized chromatin and in perturbations of the nuclear envelope, in a similar fashion as found in animal cells [41,42,43]. The behavior of *Dictyostelium* lamin during mitosis was also similar to animal cells, although *Dictyostelium* exhibits semi-closed mitosis. In animal cells, the CDK1 phosphorylation of lamins at the CDK1 consensus sequence upstream of the rod domain triggers their disassembly as a prerequisite for nuclear envelope breakdown [44]. Since the process of karyokinesis in *Dictyostelium*, i.e., separation of the two daughter nuclei, requires a deep constriction of the nuclear envelope, the nucleus needs to be pliable at the onset of mitosis. This is obviously achieved through disassembly of the lamin network at the nuclear envelope, while the lamin itself remains bound to the nuclear envelope via its isoprenyl anchor. FRAP (fluorescence recovery after photobleaching) experiments revealed a transition of low lamin mobility to high mobility at the onset of mitosis, while the protein remains associated with the nuclear envelope, obviously via its isoprenyl anchor [11]. As the timing of these events correlates with the known activity of CDK1, we analyzed the role of a serine residue within the conserved CDK1 phosphorylation site in the assembly state of *Dictyostelium* lamin [38]. As expected, and in agreement with mammalian lamins we could show that replacement with alanine (non-phosphorylatable) favored the assembled state, whereas replacement with glutamate (phosphomimetic) favored the disassembled state. Our FRAP experiments had shown that NE81 remains located at the nuclear envelope irrespective of its assembly state. In order to facilitate an analysis of the assembly state, we created mutant variants lacking the nuclear localization sequence and the C-terminal CaaX-box (NE81-∆), i.e., the prenylation signal. This NE81 variant, equipped with various tags, exhibited the assembly into large globular structures that disassembled at the onset of mitosis and were re-assembled at mitotic exit, which is in accordance to the time course of CDK1 activity [37,38,45].

The knockout of NE81 additionally produced aberrant centrosome numbers and disrupted the attachment of the centrosome to the nucleus. The latter was expected due to the linkage of the cellular cytoskeleton to the nuclear lamina via the LINC complex in many eukaryotes [46]. The LINC complex bridges the perinuclear space between the two nuclear membranes through a direct interaction between a KASH-domain protein in the outer nuclear membrane and a SUN-domain protein in the inner nuclear membrane, which interacts with the lamin meshwork [5]. These linkages also explain why the nucleus has turned out to serve as an abutment against mechanical forces acting on the whole cell [47]. In this context, the nuclear lamina is also central to the phenomenon called mechanotransduction, i.e., the conversion of mechanical stimuli into biochemical signals to induce downstream cellular responses, such as altered gene expression or cellular organization [48]. Mechanotransduction involves force transduction via focal adhesions and other cell contacts. These forces are also transduced to the nuclear surface, since the various cell contacts are connected to all three cytoskeletal elements, i.e., actin filaments, intermediate filaments, and microtubules, which in turn are directly or indirectly bound to LINC complexes at the nuclear envelope [5,49]. Mechanical stimuli, such as substrate stiffness or cell compression, are also capable of regulating the assembly state of lamins [50,51,52], which in turn are involved in the epigenetic regulation of gene expression [48]. In general, animal cells respond to increased mechanical forces with an increased level of lamin A/C, which renders the nucleus stiffer and less deformable (reviewed in [47,48,53]). Cellular tension and nuclear stiffness are also regulated by phosphorylation, which results in a reduced lamin assembly state [54,55,56]. How mechanical forces affect phosphorylation of the nuclear envelope proteins remains largely unresolved. Research on nuclear mechanics has focused mainly on mechanotransduction events in which gene expression patterns respond to mechanical forces but less on the pathways by which mechanical influences affect the assembly state of lamins.

**Figure 2:**
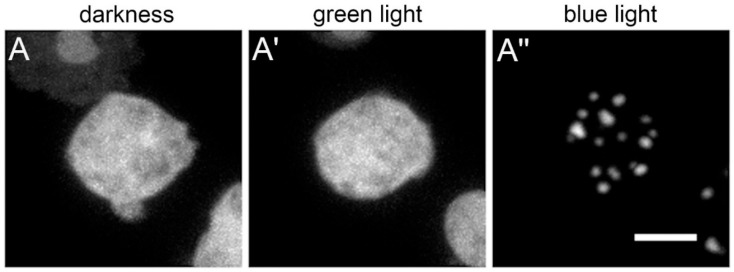
Example of blue-light-induced GFP-NE81-S122E-Δ assembly. The protein is dispersed in the cytoplasm in darkness (**A**) or upon green light exposure (**A′**) then assembles into clusters upon blue light exposure (**A″**). Bar, 5 µm. Image taken from Grafe et al. (2020) [38].

Lamin is also involved in mechanotransduction in *Dictyostelium*. This became apparent only accidentally, when phosphomimetic and non-phosphorylatable mutants of *Dictyostelium* lamin were analyzed. The GFP-NE81-S122E-∆ variant (without NLS, without CaaX-box and with a phosphomimetic point mutation at the CDK1 target site S122) exhibited an unexpected behavior in fluorescence microscopy upon GFP excitation. During imaging, the fusion protein assembled into cytosolic clusters, similar to the NE81∆ variant lacking the phosphomimetic mutation [38] (Figure 2). Clusters disassembled again in darkness. Assembly could be induced with blue but not green or near ultraviolet light and was independent of the fusion tag. Assembly similarly occurred upon cell flattening, i.e., upon mechanical stress. Earlier reports and our own observations suggested that both blue light and cell flattening resulted in a decrease in the intracellular pH. Indeed, keeping the cells at a low pH also reversibly induced cluster formation. Our results indicate that lamin assembly can be induced by various stress factors and that these are transduced via intracellular acidification. However, in the case of mechanical stress, it cannot be excluded that stretch-activated ion channels play a role as well. Such channels can be activated by stretching the lipid bilayer itself (e.g., TRAAK and TREK1) or by tension acting on the actin cytoskeleton via cell surface β1 integrins (e.g., TRPV4) [53].

The activation of these channels increases intracellular Ca^2+^, which acts as a second messenger for further signal transduction processes. Ca^2+^ signaling in the course of mechanotransduction has also been shown to be important during cell motility in both the amoeboid and multicellular states of *Dictyostelium* [57,58,59]. This involves both calcium release from the ER via the IP3 receptor and Ca^2+^ influx from the extracellular space. Although lamin assembly triggered by cell flattening has been shown in the GFP-NE81-S122E-∆ mutant, it could well play a role in the assembly of wild-type lamin as well. The *Dictyostelium* lamin mutants lacking both the NLS and CaaX-box (NE81-∆) were also very useful to prove the capability of this lamin of forming filaments in vitro. Independent of the tag, NE81-∆ mutants reversibly assembled into cytosolic clusters [37,45]. Similar to animal lamins, HisMyc-tagged NE81-∆ was highly soluble at high salt conditions and could easily be purified via affinity chromatography, while it formed assemblies under low salt conditions as shown through superresolution microscopy (both expansion microscopy and STED microscopy). Electron microscopy revealed a diameter of FLAG-tagged lamin filaments (without deletions) and of HisMyc-NE81-∆ filaments in the range of 10 nm, i.e., the diameter of cytosolic intermediate filaments, and clearly thicker than lamin filaments in MEFs, which have a diameter of only around 3.5 nm [60]. Overall, the appearance of *Dictyostelium* lamin filaments was very reminiscent to that of animal lamins in comparable assays. In addition to its structural properties and cell-cycle-dependent dynamics, it has been confirmed that the *Dictyostelium* lamin is a true lamin and not just a lamin-like protein also based on its capability to not only localize to the nuclear envelope when expressed in mammalian cells but also to partially rescue lamin mutations in MEFs. In these cells, the expression of *Dictyostelium* lamin restored nuclear circularity, reduced nuclear deformability, and prevented nuclear envelope ruptures [61]. However, in cells lacking lamin A/C, it was unable to restore the normal distribution of metazoan lamin interactors, such as emerin and nuclear pore complexes.

*Dictyostelium* lamin also behaves like a true lamin with regard to its known interaction partners. For example, it showed interactions with the HeH-family (LEM-family) protein Src1, both in a BioID-assay and in a mis-localization assay [45]. BioID was also used to demonstrate an interaction with the LINC complex protein Sun1 [62]. Taken together, based on these properties of its lamin, *Dictyostelium* has now become a useful model for the study of lamin functions [36,39,40].

### 2.2. Linker of the Nucleus and the Cytoskeleton

A key to our understanding of the mechanochemical properties of cells came with the insight that KASH-domain proteins of the outer nuclear membrane and SUN-domain proteins of the inner nuclear membrane are capable of forming the LINC complex (linker of the nucleus and the cytoskeleton), which forms a bridge connecting the cytosolic cytoskeleton (i.e., actin filaments, microtubules and intermediate filaments) with the nuclear lamina [5]. SUN is an acronym derived from the names of the first identified members of this family of nuclear membrane proteins, *Schizosaccharomyces pombe* Sad1 [63] and *C. elegans* UNC-84 [64]. They are characterized by a common C-terminal SUN-domain, which interacts with the C-terminal KASH-domain of their counterparts within the perinuclear space. The acronym KASH was derived from the names Klarsicht, ANC-1, and Syne homology, i.e., the founding members of this family of often very large membrane proteins, which usually also contain spectrin repeats (reviewed by [65]). Both SUN- and KASH-domain proteins occur in several isoforms. While mammalian cells contain two SUN proteins, SUN1 and SUN2, expressed in all tissues, and three further testis-specific isoforms, they express up to six KASH-domain proteins, i.e., nesprin-1, -2, -3, -4, CCDC155, and Jaw1/LRMP. Meanwhile they are also called KASH1-6 (reviewed by [65]). SUN-domain proteins were originally classified as type II single pass membrane proteins. Yet, using a synthetic biology platform for the reconstitution and mechanistic dissection of LINC complex assembly, Luxton and co-workers have shown that the real situation is more complex [66]. In artificial nuclear membranes, SUN1 contains three transmembrane domains and a hydrophobic domain with a partial membrane pass, while SUN2 contains only one transmembrane domain and also a partial membrane pass through the inner nuclear membrane. However, in both cases, the C-termini, including the SUN-domains, are oriented towards the perinuclear space.

SUN-domain proteins generally tend to form stable trimers, which in turn interact with three chains of KASH-domain proteins [46,67]. The stability of this interaction can be further increased by intermolecular disulfide bonds [68]. LINC complexes are rather variable in composition due to the ability of SUN-domain proteins to form heterotrimers and the ability of SUN-complexes to interact with various KASH-domain proteins [65]. In addition to the canonical hetero-hexamer consisting of each three SUN and three KASH protein chains, SUN1 by itself can also form hexamers, by head-to-head interaction of the SUN domains between two trimers [67,69,70,71]. Cruz and co-workers suggested that this so-called apo-SUN structure may represent an autoinhibited state during transport from the ER to the inner nuclear membrane [67]. At the nuclear membrane, SUN and KASH domain proteins may form even higher-order networks in response to mechanical forces [70,72].

**Figure 3:**
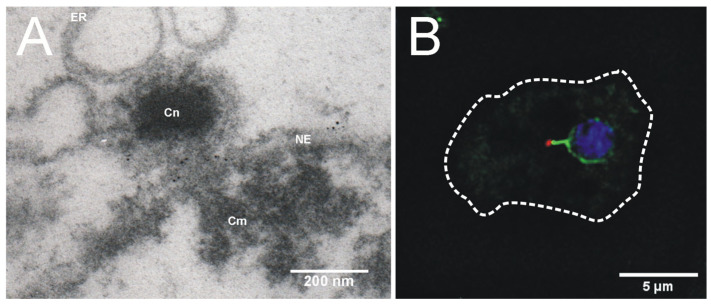
Centrosome–nucleus–centromere cluster. (**A**) Immunoelectron microscopy image showing one section of an isolated nucleus with the attached centrosome. Nuclei were labeled with an antibody against *D. discoideum* Sun1 and nanogold conjugated anti-rabbit antibodies. The centrosome (Cn), the centromeric cluster (Cm), the nuclear envelope (NE), and the endoplasmic reticulum (ER) are indicated (image by Prof. Otto Baumann). (**B**) Immunofluorescence microscopy image of a Sun1-GFP-knock-in cell (green) stained with an antibody against the centrosomal core protein CP91 and anti-rabbit-AlexaFluor 568 conjugates (red) and DAPI (blue). The cell edges are outlined by a dashed line. Taken from Batsios et al. (2019) [78].

*Dictyostelium discoideum* expresses two SUN-domain proteins, Sun1 [6,7], as described in more detail below, and SunB [73]. The latter plays a role in development in a transcriptional signaling pathway, but a function in linking the cytoskeleton to the nucleus has not been shown. Thus, SunB is certainly not an orthologue of mammalian SUN2. In contrast, Sun1 is clearly required for anchoring the centrosome to the nucleus and is related to cytoskeletal functions. Antibodies against *Dictyostelium* Sun1 strongly stained the pericentrosomal region of the nuclear envelope and a bright spot at the centrosome. The same holds true for Sun1-GFP if expressed as a replacement of endogenous Sun1 via a knock-in construct (Figure 3). Furthermore, FRAP experiments in cells expressing GFP-tagged Sun1 revealed the existence of two populations at the nuclear envelope, a rather immobile one in the pericentrosomal region and an adjacent, more mobile population at the nuclear envelope [6]. All mutations interfering with Sun1 functions caused a detachment of the centrosome from the nuclear envelope, and frequently there were aberrant centrosome numbers. Not surprisingly, this phenotype was similar to the phenotype observed upon interference with lamin functions in *Dictyostelium*, as Sun1 and lamin interact in BioID experiments [62]. With regard to putative KASH-domain proteins involved in linking the cytoskeleton to the nucleus, the situation is unclear, since the *Dictyostelium* genome encodes no readily identifiable KASH-domain protein. Clearly, there is a spectrin-superfamily protein, called interaptin, which is associated with the outer nuclear envelope and interacts with actin [1,7]. Interaptin-null cells are viable and affected mainly in development [1,74]. Whether interaptin really is a functional representative of nesprins in *Dictyostelium* remains questionable, since its short C-terminus contains no clearly identifiable KASH-domain. Furthermore, its staining pattern at the nuclear envelope does not correspond exactly to that of Sun1, and further data suggested that it does not bind to Sun1 [7]. Moreover, it is certainly not involved in tethering the centrosome to the nucleus.

Various experimental data indicate that centrosome–nucleus attachment in *Dictyostelium* depends on several, possibly unrelated processes. The most likely representative of something like a LINC complex is a complex consisting of Sun1 and an unusual kinesin, Kif9 [8,9]. Kif9 is an internal motor kinesin of the kinesin-13 family. Like other so-called internal motor kinesins, Kif9 possesses an N-terminal SxIP domain, which is thought to regulate interactions with microtubule tip-binding proteins [8]. As a rather unique feature, it has a ~23 residue, C-terminal transmembrane domain anchoring it at the nuclear envelope. When expressed as a GFP-fusion protein, Kif9 concentrates in the pericentrosomal region of the nuclear envelope. This pattern is also observed when the transmembrane domain alone fused to GFP is expressed alongside endogenous Kif9. However, when expressed in a Kif9-null background in *Dictyostelium* or in HeLa cells (lacking a comparable kinesin), the transmembrane domain is evenly distributed across the nuclear envelope [8]. The pericentrosomal localization of full-length GFP-Kif9 was similar to Sun1 staining. This and the fact that the knockout of Kif9 resulted in a redistribution of Sun1, from the pericentrosomal localization pattern towards an even distribution across the nuclear envelope, prompted the idea that Sun1 and Kif9 could interact to form a LINC-like complex, despite the lack of a clear KASH domain at the Kif9 C-terminus [8]. Due to this and the microtubule-binding activity of Kif9, Tikhonenko and co-workers hypothesized that Kif9 engages microtubules via its motor domain and uses its microtubule-depolymerizing activity to exert a directed pulling force on the centrosome towards the nuclear envelope. This scenario was also supported by the observation that Kif9-null cells display centrosome detachment from the nuclear envelope during interphase and multiple mitotic perturbations [8]. The cooperation of SUN proteins and kinesins is not without precedent, although so far these interactions have not been shown to play a role in centrosome–nucleus attachment in other organisms [75]. Several KASH proteins, including mammalian nesprin-4, *C. elegans* UNC-83, and *Drosophila* Klarsicht, bind to a kinesin light chain to target kinesin-1 to the outer nuclear membrane [76,77].

Another scenario for centrosome linkage to the nucleus is that Sun1, located in the outer nuclear membrane, could bind to the centrosome by itself and link it via an interaction with Sun1 in the inner nuclear membrane, either directly or via a further partner within the perinuclear space. This hypothesis may sound unusual, having the classical model in mind, which was originally based on results in *C. elegans*. Here SUN-1 in the inner nuclear membrane binds to the KASH-domain protein ZYG-12 in the outer nuclear membrane. ZYG-12 in turn binds to dynein in the cytosol, to reel the centrosome in towards the nucleus via attached microtubules [79]. The idea of a Sun1–Sun1 interaction in centrosome–nucleus attachment is supported by the observation that antibodies against Sun1 always stain a bright spot directly at the centrosome itself that is bridged via a thin connection to the nuclear envelope, which is strongly stained only in the pericentrosomal region. Moreover, Sun1 co-purified with isolated centrosomes and immunogold electron microscopy showed that Sun1 is equally distributed in both nuclear membranes [6]. Having in mind also the capability of SUN proteins forming hexamers via a head-to-head interaction of the Sun domains of two trimers (see above; [67]), it is well possible that centrosomes are attached to the nuclear envelope via a hexameric Sun1 complex.

In addition to Kif9, a further motor protein involved in centrosome attachment to the nucleus appears to be dynein. This came from the observation that the expression of a hypomorphic mutant of the dynein regulator LIS1 caused centrosome detachment from the nucleus [80]. LIS1 binds to the dynein heavy chain, a fraction of dynein was detected at the nucleus, and both were detected also at centrosomes [80]. Thus, it is possible that nuclear envelope-associated dynein employs its microtubule minus-end-directed motor activity to bring the centrosome close to the nucleus. This process could be assisted by the microtubule-binding and depolymerizing activity of Kif9. Although an interaction of dynein/LIS1 with Sun1 has not yet been proven with a protein–protein interaction assay, the observation that the pericentrosomal localization of Sun1 requires intact DdLIS1 [81] suggests at least an indirect interaction between these proteins.

The centrosome detachment phenotype was also observed upon depletion of the centrosomal corona protein CP148 [82], as well as upon knockout of the centrin-family protein CenB [83]. However, in both cases, it remains unknown how these proteins could participate in centrosome/nucleus attachment. No interaction with Sun1 or other known putative partner proteins at the nuclear envelope has been shown. The same applies to another centrosomal corona protein, CP248/CP250 [35,84]. Here, knockout of the protein resulted in a reduced amount of both Sun1 and interaptin at the nuclear envelope, although a direct interaction between these proteins is unlikely [84]. The centrosome remains associated with the nucleus even after loss of the corona upon centrosome duplication in prophase, which also points to a role of centrosomal core layer proteins in this process.

### 2.3. Nuclear Envelope Transmembrane Proteins at the Interface of the Centrosome and the Centromere Cluster

**Figure 4:**
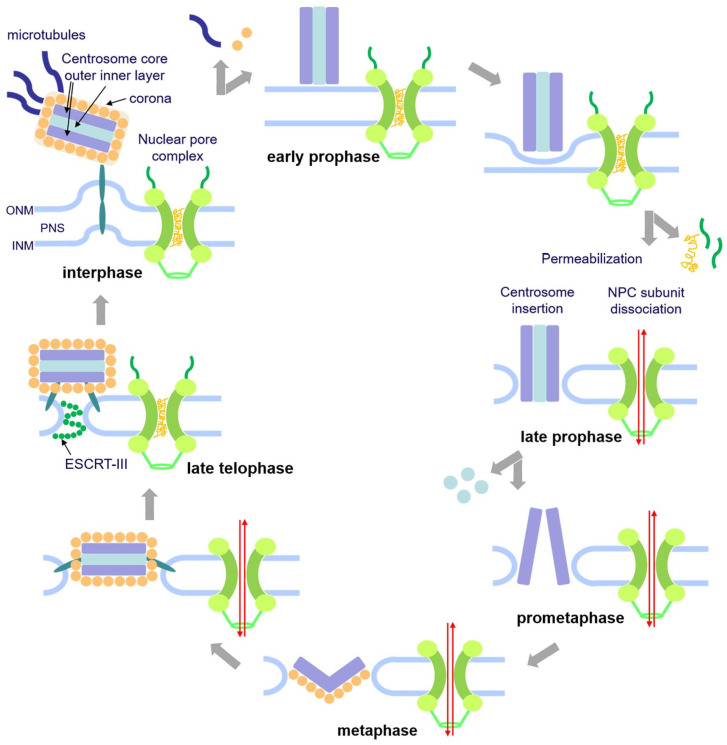
Model of centrosome insertion and nuclear envelope permeabilization in *Dictyostelium discoideum*. The onset of mitosis is characterized by the loss of the corona together with the attached microtubules in early prophase. The fusion of the inner and outer nuclear membranes forms a fenestra and allows the insertion of the mitotic centrosome into the nuclear envelope. Simultaneously, the partial disassembly of the nuclear pore complex contributes to the permeabilization of the nuclear envelope in late prophase. Next, in prometaphase, the central layer of the core structure disappears, the mitotic centrosome splits, and the outer entities move apart. During telophase, the central core layer re-appears and the nuclear pore complexes get reassembled. The duplicated centrosomes exit their fenestrae in the nuclear envelope, and the ESCRT-III complex starts re-sealing the nuclear envelope to close the fenestrae. Taken from Mitic et al. (2023) [12].

Obviously, the Sun1-containing centrosome linker is not only responsible for holding the centrosome in place but also for clustering the centromeres in the pericentrosomal region of the nuclear interior (Figure 3). Thus, interference with Sun1 functions is accompanied by the displacement of centromeres from the pericentrosomal region [6]. This phenotype was also observed upon centrosome detachment in an RNAi strain with the depletion of CP148 [82] and upon the knockout of Kif9 [8]. This underscores the idea that Sun1 is a key component of the physical connection between centrosomes and centromeres, spanning both nuclear membranes. The centromere cluster is observed throughout the entire interphase and stable until prometaphase. It is likely that centromere clustering has evolved to facilitate spindle formation in *Dictyostelium*. In prophase, the centrosome inserts into the nuclear envelope and duplicates [12] (Figure 4). The close vicinity of the centromere cluster greatly facilitates the establishment of microtubule connections between the mitotic centrosomes and the kinetochores, which are assembled at the centromeres. This may be viewed as either a reason or a consequence of the obvious absence of acentrosomal pathways for bipolar spindle assembly (for a review on spindle assembly see [85]). These would require the Ran-GTP-dependent activation of spindle assembly factors, first of all TPX2 and aurora A kinase. Yet, no TPX2 orthologue appears to exist in *Dictyostelium,* and there is no evidence for a role in spindle formation for AurK, the only aurora kinase in this organism [86]. The situation in *Dictyostelium* is very reminiscent of that in fission yeast, where the spindle pole body (yeast centrosome) is associated with the cytosolic side of the nucleus during interphase and centromeres are clustered adjacent to the spindle pole body as well [87]. Here, the LINC complex connects the spindle pole body with the centromere cluster across the nuclear envelope [88]. Initially, it was claimed that centromere clustering is mediated by the inner nuclear membrane protein Ima1, together with the Ndc80 complex of the outer kinetochore [87]. Ima1 belongs to a family of inner nuclear membrane proteins identified in many metazoans and fungi, including Net1 and Samp1 [89,90,91]. The latter was initially identified through subtractive proteomics and later found to associate with membrane compartments along the mitotic spindle (hence the name spindle associated membrane protein 1). In animal cells, Samp1 is associated with the LINC complex, lamin A/C, and the LEM-domain protein Emerin and is engaged in peripheral heterochromatin organization [92,93]. However, the initial study in fission yeast was challenged by a later one, where Ima1 was shown to be dispensible for centromere clustering in fission yeast [94]. Rather, Ima1 cooperates with HeH/LEM-domain proteins (see above) to maintain nuclear envelope organization. The *Dictyostelium* genome contains a close homologue of Ima1 (encoded by DDB_G0292450).

The GFP-tagged Ima1 protein localizes to the nuclear envelope, but otherwise, it remains poorly characterized (Denis Larochelle, personal communication). Yet, according to the results in fission yeast, it is not a promising candidate for a role in centromere clustering. The current view for fission yeast is that centromere clustering adjacent to the SPB involves Csi1, which binds to both centromeres and the SUN-domain protein Sad1 [95] and to the HeH/LEM-domain protein Lem2 [96].

**Figure 5:**
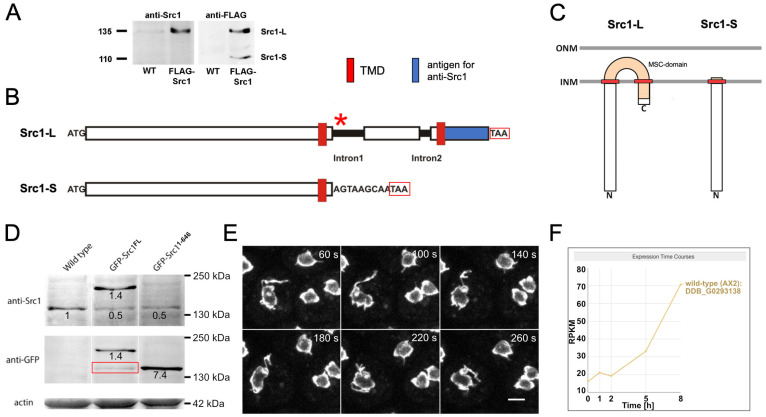
The INM protein Src1 in *Dictyostelium* has two isoforms and is upregulated during development. (**A**) Western blot analysis of whole cell lysates of the *Dictyostelium* wild-type (WT) and a strain expressing Src1 fused to a FLAG-tag at its N-terminus (FLAG-Src1). For this, GFP in the GFP-Src1^FL^ construct [45] was replaced by a FLAG tag. Two discrete FLAG-tagged proteins (anti-FLAG) encoding two isoforms, Src1-L and Src1-S, are visible. The blot was incubated either with anti-Src1 (against the C-terminus) or anti-FLAG (Invitrogen) and a secondary alkaline phosphatase conjugated antibody and stained with NBT/BCIP. (**B**) Predicted variants of the Src1 gene with 3170 bp, including two introns (251 bp, 88 bp): Src1-L (long isoform), generated through constitutive splicing, has a 2829 bp coding sequence resulting in 942 amino acids and a molecular mass of 107 kDa. The Src1-S (short isoform), generated via intron retention, has a 1950 bp coding sequence encoding a protein with 649 amino acids and a molecular mass of 73 kDa. TAA stop codons are indicated by the red asterisk and red boxes, respectively. (**C**) Membrane topology of Src1-L and Src1-S in the nuclear envelope. Src1-L has two transmembrane domains (TMD) spanning the INM, a MSC-domain inside the perinuclear space, and a C-terminus facing the nuclear interior. Src1-S possesses only one TMD and is lacking the MSC domain. (**D**) Western blot from Batsios et al. 2016 [45]. GFP-Src1^FL^ cells stained with anti-GFP antibodies likewise showed a second GFP-tagged protein encoding the short isoform (middle row, red rectangle). (**E**) Selected time points of cells overexpressing the GFP-Src1^FL^-fusion protein imaged with a confocal spinning disk microscope. Cells show motile nuclear nozzles reminiscent of that found in cells in early developmental stages. Bar, 5 µm. (**F**) The expression of Src1 (DDB_G0293138) mRNA is upregulated during early development stages (hours 2–8). RPKM (Reads per kilo base per million mapped reads) are plotted against time (hours). Expression time courses acquired from dictyExpress (https://app.dictyexpress.org/bcm/, accessed on 22 January 2025 [101]).

While no orthologue of Csi1 appears to exist in *Dictyostelium*, there is a gene encoding a single helix-extension-helix (HeH)-family protein, called Src1 [45]. This protein family of inner nuclear membrane proteins was originally called the LEM-domain family, in reference to a common conserved bihelical motif found in the original members LAP2, Emerin, and MAN1. There is evidence that the LEM domain has evolved from a more ancient bihelical motif, the HeH motif, found in the nuclear membrane proteins of all eukaryotic phyla [97]. There are indications that the LEM domain co-evolved with the emergence of BAF (barrier to autointegration factor), which mediates the association of the LEM domain with DNA. Hence, organisms naturally lacking BAF also lack proteins with a *bona fide* LEM domain, instead they possess proteins with a LEM-like domain or HeH domain, both of which are able to bind to DNA directly. HeH-family proteins contain either one (e. g. LAP2, Emerin) or two transmembrane domains (e. g. MAN1). HeH-family proteins in non-metazoan organisms lack the LEM domain, typically include two transmembrane domains, and their N- and C-termini both extend into the nucleoplasm (Figure 5C). Both in budding yeast and fission yeast, there are two genes encoding HeH-family proteins with two transmembrane domains, i.e., Lem2 and Man1 in *S. pombe* [98] and Src1 (also known as Heh1) and Heh2 in *S. cerevisiae* [99]. Furthermore, *S. cerevisiae HEH1* yields two isoforms due to alternative splicing, a long one (Heh1-L) with two transmembrane domains and a shorter isoform (Heh1-S) with only one transmembrane domain, which are both required for the ESCRT-mediated repair of NE damages [100] (see also below). The *Dictyostelium* genome encodes only one Heh-family protein, Src1. However, unlike other proteins of the HeH family, *Dictyostelium* Src1 lacks the canonical HeH domain, while otherwise, it clearly groups with the HeH family, due to its similarity to MAN1-like proteins [45,97]. As in *S. cerevisiae HEH1*, *Dictyostelium Src1* appears to encode two variants of the protein, one long, Man1-like isoform with two transmembrane domains (Src1-L), and a C-terminally truncated variant with only one transmembrane domain (Src1-S), which is more reminiscent of LAP2. This became apparent in a strain expressing Src1 with an N-terminal FLAG tag and an N-terminal GFP tag (Figure 5). Since the plasmid encoding the protein contained the complete genomic sequence, including introns, processing of the pre-mRNA was required. The expression of two discrete FLAG/GFP-tagged proteins, obviously representing two distinct isoforms, was demonstrated through Western blotting (Figure 5A; Batsios, previously unpublished).

Staining behavior with an anti-Src1 antibody directed against the C-terminus of the full-length isoform suggests that the truncated isoform contains only one transmembrane domain, while the full-length protein contains two (Figure 5A,D). It is likely that the truncated isoform results from retention of the first intron, which is located downstream of the sequence encoding the first transmembrane domain, and includes a stop codon (Figure 5B). Yet, although distributed across the whole nuclear envelope, both isoforms were concentrated adjacent to the nucleoli, which are attached to the nuclear envelope (Figure 1A). To date, no differential functions of the two isoforms are known. The strong co-localization of Src1 with nucleoli suggests a nucleolar function, which would not be without precedent for HeH proteins. In budding yeast, Src1 is involved in the stabilization of highly repetitive rDNA sequences at the nuclear periphery, in cooperation with other proteins [102]. The cell-cycle regulated Man1 of *Schizosaccharomyces japonicus* appears to be required for nucleolar disassembly by regulating the condensation of rDNA arrays [103].

A further function of HeH-family proteins is to associate with heterochromatin and promote its formation. In fission yeast, the N-terminal part of Lem2 containing the conserved HeH domain mediates binding to centromeres and their clustering, while telomeric localization and the silencing of heterochromatic domains both exclusively depend on the conserved MSC (MAN1-Src1 C-terminal) domain [96]. Whether *Dictyostelium* Src1 is involved in centromere clustering is unknown. However, during late mitosis, it is slightly enriched in the pericentrosomal area and at the abscission site during karyokinesis, indicating a role in centromere clustering and abscission. A role in abscission during karyokinesis in *Aspergillus nidulans* was also discussed. Here, Src1 is required for proper abscission and stable post-mitotic G1 nuclear formation [104]. A corresponding role in *Dictyostelium* is supported by the observation that Src1 co-localizes with the microtubule-severing protein DdSpastin and interacts with it in proximity-dependent biotin identification (BioID) experiments (see below; [19]). BioID also revealed an interaction with the *Dictyostelium* lamin [45], another parallel to MAN1 in animal cells. The binding of Src1 to lamin appears to not be obligatory. Although both proteins co-localize at the nuclear envelope in vegetative cells during interphase and mitosis, there are conditions in which Src1 localizes to nuclear membrane regions without co-localizing with lamin. This was initially observed when the N-terminal 646 amino acids of Src1 (including the first transmembrane domain, i.e., corresponding to the short isoform) were overexpressed as a GFP-fusion protein [45]. Under these conditions, the nuclear envelope displayed one to three thread-like extensions into the cytoplasm, called nuclear nozzles, which were labeled by the GFP fusion protein. The same phenotype was observed when full-length GFP-Src1 was overexpressed (Figure 5E). Nozzles typically appeared at sites adjacent to nucleoli, and their presence depended on microtubules, which prompted us to suppose that they appear at sites where the outer nuclear envelope interacts with microtubules. The nozzles themselves were labeled by the inner nuclear membrane protein GFP-Src1 but did not co-localize with lamin, suggesting that they were composed of both nuclear membranes and had lost contact with the nuclear lamina underneath. Interestingly, this phenotype mimicked a situation that occurs naturally during the aggregation phase of *Dictyostelium* development from an amoeboid state to a multicellular fruiting body. During this process, the nuclei change from a spherical toward an elongated, ellipsoid shape. At the same time, the two to three separate nucleoli unite into a single nucleolus at the apex of the ellipsoid nucleus, opposing the apex where the centrosome is attached. Sameshima and co-workers have shown that nuclear nozzles appear at this developmental stage at sites associated with the single nucleolus [21,105]. These nuclear nozzles were highly reminiscent of those observed upon GFP-Src1 overexpression in vegetative cells. Knowing that during aggregation Src1 expression increases approximately 5-fold compared to vegetative cells (Figure 5F; DictyExpress database; [101]), it is likely that the appearance of nuclear nozzles is actually driven by the increased expression of Src1. The significance of nuclear nozzle formation remains to be investigated.

### 2.4. A Role of Lem/Heh-Domain Proteins in Mitotic Nuclear Envelope Remodeling

A further role of LEM/HeH-domain proteins during late mitosis appears to be conserved in *Dictyostelium* as well. In animal cells with open mitosis, the nuclear envelope re-forms during telophase [106]. Yet, at that time, the mitotic spindle, including kinetochore microtubules and pole-to-pole microtubules, has not yet disassembled, and thus, these microtubules sterically interfere with the closure of gaps in the nuclear envelope, both at the two poles and around the central spindle. The closure of these gaps requires the local disassembly of microtubules [107]. This is driven by the AAA-ATPase SPASTIN, whose recruitment in turn requires at least the ESCRT-II/III-like protein CHMP7, IST1, and the LEM-domain protein LEM2/LEMD2 [108,109,110,111]. In this pathway, which is also conserved in yeast, CHMP7 and LEMD2 play a key role in the closure of holes in the nuclear envelope. In case of intact nuclei, both proteins are differentially localized with LEMD2 at the inner nuclear membrane and CHMP7 in the cytosol. In the case of discontinuities of the nuclear envelope, as for example upon ruptures or in late mitosis, both proteins come together and form a complex at the hole, which recruits further ESCRT components to close the hole [110,112]. Despite the semi-closed mitosis in *Dictyostelium*, the condition of the nuclear envelope in late telophase is very similar, in that there is an almost continuous nuclear envelope, which displays gaps around the spindle microtubules. *Dictyostelium* also possesses an orthologue of SPASTIN (DdSpastin), which binds to microtubules, severs microtubules, and accumulates at the gaps, both at the poles and at the abscission site of the separating daughter nuclei around the central spindle [19]. These properties are in agreement with a role of DdSpastin in the severing of spindle microtubules that would otherwise interfere with closure of the fenestrae. The concept of a conserved principal pathway of mitotic nuclear envelope remodeling between animals and *Dictyostelium* amoebae is also corroborated by the fact that DdSpastin interacts with the HeH/LEM-family protein Src1 in BioID assays, as well as with Sun1. It also co-localizes with Src1, Sun1, the ESCRT component CHMP7, and the IST1-like protein filactin. Yet, there appear to be differences in the mechanism of how fenestrae are closed at the poles and the abscission sites, respectively. Filactin co-localized with DdSpastin only at the abscission sites and was absent from the poles, while Sun1 showed the opposite behavior, meaning that Sun1 was absent from abscission sites but concentrated at the poles [19]. Here, Sun1 formed a collar around the mitotic centrosomes, which at this point, were still inserted in the nuclear envelope. Interestingly this collar-like distribution at the centrosome fenestrae was also found for the NPC proteins NUP210 and TPR [18]. Although so far there is no experimental evidence, this suggests a further role of NPC proteins in the closure of the mitotic nuclear envelope fenestrae at the poles in *Dictyostelium*. Overall, the situation appears similar to that in fission yeast where the Sun1 orthologue Sad1 together with an NPC component and other proteins forms a ring-like structure around the spindle pole body (SPB) during the process of SPB insertion [113] (see below). There are also some similarities in the process of SPB extrusion from the nuclear envelope in late mitosis to the analogous process in *Dictyostelium*. In fission yeast, the extrusion process starts in anaphase B when Lem2/Heh1 (see above) recruits Cmp7, which together with SPB proteins forms a proteinacious diffusion barrier to prevent diffusion through the still open fenestra at the SPB attachment site [114]. This triggers the recruitment of Ist1 and of further ESCRT components, which then mediate the constriction of the fenestra and form a further sealing grommet around the SPB. Finally around the time of G1/S transition, after the recruitment of ESCRT subunits and the displacement of Cmp7, the nuclear envelope becomes sealed [114]. Compared to fission yeast, in *Dictyostelium*, the pathway of nuclear envelope sealing at the poles has not been fully characterized. As in animal cells and fission yeast, a HeH-family protein and CHMP7 certainly participate in the process. However, there are several differences to fission yeast. First, no sealing grommet is required at the interface between the mitotic centrosomes and the fenestrae of the NE, since mitosis is semi-closed and the permeability barrier of the nuclear envelope is restored only in the next S-phase, shortly after cytokinesis (there is no apparent G1 phase in *Dictyostelium* [115]) [12]. Second, DdSpastin appears to be an important player in nuclear envelope dynamics during late mitosis by ensuring the cleavage of microtubules that would sterically interfere with the closure of nuclear envelope holes (see above) [19,108]. In contrast, no spastin-like protein seems to be involved in fission yeast. Third, the Ist1-like protein filactin was found only at the abscission sites of the nuclear envelope during karyokinesis but not adjacent to centrosomes at the poles. Further research is necessary to understand how these differences can be explained by the mode of mitosis (closed vs. semi-closed) and whether the absence of a permeability barrier during mitosis implies a higher similarity of nuclear envelope restoration between cells with an open or semi-closed mitosis.

### 2.5. Nuclear Pore Complexes

Already about two decades ago, the NPC of *Dictyostelium* was studied extensively on a structural level employing cryo-electron tomography [2,4]. As expected, it showed a similar overall structure as that of animal cells or yeast [116]. NPCs are composed of a ring consisting of transmembrane nucleoporins (NUPs), which are associated with an inner ring and two outer rings, one on the cytosolic side and one on the nuclear side. On both sides, the outer rings are composed of the Y-complex consisting of seven conserved proteins [117]. The cytoplasmic outer ring is associated with cytoplasmic filaments, while the nuclear outer ring is associated with the nuclear basket. The inner ring is bound to FG-repeat nucleoporins (rich in phenylalanine-glycine repeats), which form a hydrogel required for transport via a phase separation mechanism [118]. Most of the nucleoporins occur in a multitude of eight, which is explained by the eight-fold radial symmetry of the NPC. Most of the about 30 known nucleoporins are conserved in all eukaryotes. The extent of nucleoporin sequence conservation varies, and thus, orthologues may differ considerably both in primary structure and molecular mass, which is also the reason why these orthologues, usually named NUP plus a number indicating the molecular mass, unfortunately have different names in different species.

**Figure 6:**
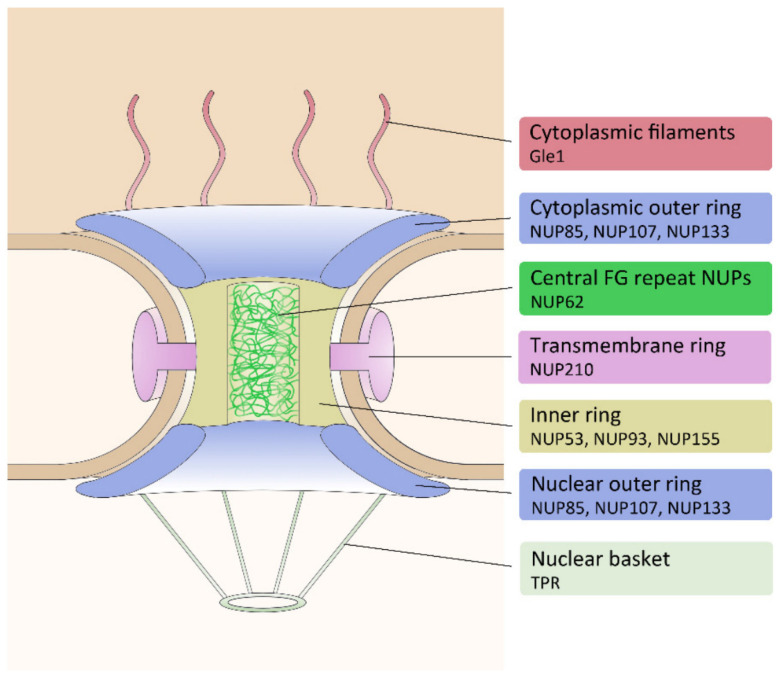
Schematic of the nuclear pore complex. Its characteristic parts and their marker proteins used in *Dictyostelium* are indicated on the right. Image taken from Mitic et al. (2022) [18].

Despite the in-depth structural characterization of the *Dictyostelium* NPC, it took until 2022 for representative members of the NPC substructures to be studied on a molecular level and for disclosure of their dynamic properties [18] (Figure 6). Meanwhile, about twenty genes encoding NUPs have been identified in the *Dictyostelium* genome. Ten representative candidates were selected and expressed as green-fluorescent fusion proteins (tagged with GFP or mNeon). Fluorescence microscopy revealed that NPCs are absent from the contact area of the nucleoli and that some nucleoporins, e.g., NUP53, are also present at the centrosome and the spindle poles. During mitosis, the central FG protein NUP62, two inner ring components, and the cytosolic filament component Gle1 are released from the NPC, while all other tested NUPs (outer ring proteins and the nuclear basket marker TPR) remained at the NE.

This situation was highly reminiscent of that observed in *Aspergillus nidulans* [17,119], where there is also a release of central FG NUPs, together with several components of the inner ring, from the pore complex, enabling the free diffusion of proteins independent of Ran-dependent-directed nuclear transport. It allows tubulin and spindle assembly factors to enter the nucleus and build up the spindle, even though they do not carry a nuclear localization sequence. Partial disassembly of the NPCs takes place also in *Dictyostelium,* leading to the conclusion that it uses the same pathway of permeabilization of the nuclear envelope during semi-closed mitosis as *Aspergillus*. This was shown using strains expressing NLS-tdTom, an NLS-tagged version of two tandem copies of the red-fluorescent protein tomato, as a marker to visualize nuclear envelope permeabilization [12]. During interphase, this marker concentrates in the nuclear interior, while it rapidly diffuses out of the nucleus exactly at the time of the dissociation of Nup62, which was used as a representative for FG-repeat nucleoporins. Interestingly, the restoration of nuclear envelope integrity (i.e., the permeability barrier) takes place long after the re-assembly of NPCs and cytokinesis and is accompanied by a concentration of endosomal sorting complex required for transport (ESCRT) components at both sites of nuclear envelope fenestration (centrosome and abscission site of daughter nuclei around the central spindle). One possible interpretation is that NPC disassembly is required for permeabilization during early mitosis.

**Figure 7:**
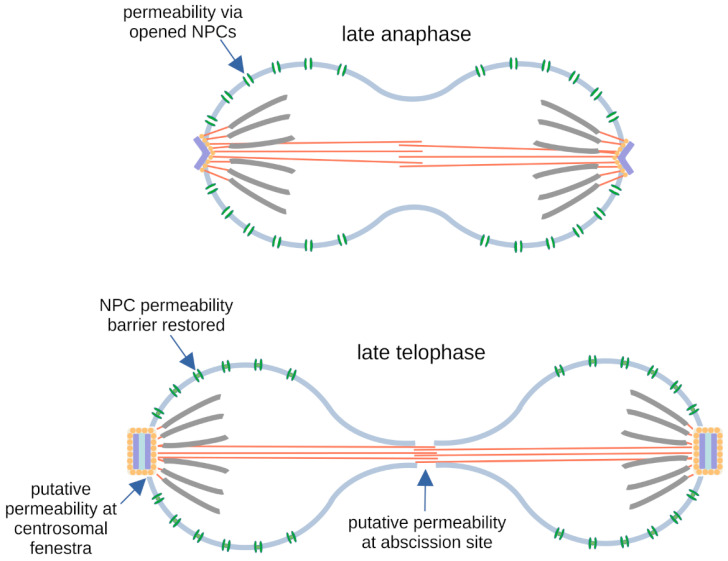
Nuclear envelope conformations during late anaphase and telophase. Situations in late anaphase and late telophase are shown. Mitotic centrosomes at the poles undergo a folding process during telophase and exit their nuclear envelope fenestrae during very late mitosis (see also Figure 4). Until early mitosis, nuclear envelope permeability is achieved via partially disassembled NPCs (green), while the nuclear membranes remain intact. In late mitosis, the nuclear envelope ruptures at the abscission site between the two daughter nuclei during karyokinesis. Putative additional sites of permeability are indicated. The chromosomes are drawn in gray, and microtubules comprising both kinetochore microtubules and the central spindle are drawn in dark orange.

Whereas the fenestrae in the nuclear envelope ensure the maintenance of mitotic nuclear envelope permeability even after NPCs are reassembled (Figure 7), of course, this raises the question of whether centrosome insertion into the nuclear envelope during prophase also participates in the permeabilization process. Yet, according to ultrastructural analyses there is no obvious space between the nuclear envelope and the embedded mitotic centrosome [120], and centrosome insertion occurs in parallel to partial NPC disassembly. Thus, mechanistically there is no need for a further permeabilization pathway in addition to partial NPC disassembly. Furthermore, unpublished results using a strain defective in centrosome insertion indicate that the mitotic release of NLS-tdTom from the nucleus occurs independently of centrosome insertion (Kristina Mitic, unpublished observation). With regard to centrosome insertion, the situation in *Dictyostelium* is somewhat reminiscent of that in *S. pombe*. As in *Dictyostelium*, the spindle pole body/centrosome also enters and leaves the nuclear envelope throughout the cell cycle, and centromeres are clustered adjacent to the centrosome also in interphase [95,121,122,123,124]. However, in *S. pombe*, SPB duplication occurs prior to the insertion process, whereas in *Dictyostelium*, it clearly occurs after insertion [12]. Furthermore, fission yeast mitosis is completely closed, although there is a controlled local nuclear envelope breakdown around the central spindle to enable karyokinesis [125,126]. Despite these differences, it is plausible that *Dictyostelium* and fission yeast employ a similar pathway of centrosome insertion and that the interface between inserted mitotic centrosomes and the nuclear envelope is tight also in *Dictyostelium*, at least during early mitosis.

In fission yeast, the Sun-domain protein Sad1, the KASH-domain protein Kms2, the mitotic regulator Cut12, and Cut11 (a component of both SPBs and NPCs), together form a ring around the SPB, for which formation depends on Sad1 and the centromere cluster [124]. The presence of the ring is required for SPB insertion and is regulated by the Polo kinase Plo1. As a further player in the insertion process, the transmembrane protein Brr6 is transiently recruited to the SPB during both the nuclear envelope insertion at the onset of mitosis and the extrusion of the SPBs during mitotic exit [127]. Brr6 genetically interacts with the ER membrane protein Apq12, which is also involved in lipid metabolism in budding yeast [127,128,129] suggesting that Brr6 alters the nuclear envelope composition at the duplicated SPBs to promote SPB insertion and extrusion. Interestingly, orthologues of Brr6 were found only in organisms in which fenestration of the nuclear envelope occurs in a process of SPB/centrosome insertion in the course of mitotic spindle organization and where a single microtubule-organizing center organizes microtubules on both sides of an otherwise closed nuclear envelope. In budding yeast, the SPB remains inserted in the nuclear envelope also during interphase. Yet, the insertion of the precursor of the new spindle pole body, the duplication plaque, employs essentially the same set of proteins, which is called the spindle pole insertion network [130,131,132].

In *Dictyostelium*, the insertion process remains to be characterized more fully. However Sun1, together with NPC proteins—especially NUP210 and TPR—also form a ring-like structure around the inserted mitotic centrosomes [6,18], the polo-like kinase Plk is concentrated at mitotic spindle poles [86], and the *Dictyostelium* genome contains a homologue of Brr6/Brl1. These are all good reasons to assume that the insertion mechanism described in yeasts is conserved in *Dictyostelium* amoebae.

## 3. Conclusions

With regard to organization of the nuclear envelope, *Dictyostelium* amoebae appear like a chimera of fungal and metazoan cells. Apart from the NPCs that are universal in all eukaryotes, they share with animal cells a lamin-based nuclear lamina and similar challenges in repairing the nuclear envelope in late mitosis. This leads to the involvement of a similar set of proteins. With fungi, on the other hand, they show more similarities at the transition from G2 phase to M phase, as they also possess acentriolar centrosomes, which, at least in some species, also insert from the cytoplasmic side into a fenestra of the nuclear envelope at the transition to mitosis, so that the mitotic spindle can be formed with otherwise intact nuclear membranes. As in many fungi, mitosis in *Dictyostelium* is semi-closed, and the access of spindle assembly factors to the nuclear interior is provided by partially disassembled NPCs. We are confident that future research on *Dictyostelium* nuclear envelope dynamics will provide further valuable insights into the early evolution of eukaryotes.

## Data Availability

No new data were created or analyzed in this study. Data sharing is not applicable to this article.

## References

[1] Rivero F., Kuspa A., Brokamp R., Matzner M., Noegel A.A. (1998). Interaptin, an Actin-Binding Protein of the Alpha-Actinin Superfamily in *Dictyostelium discoideum*, is Developmentally and cAMP-Regulated and Associates with Intracellular Membrane Compartments. J. Cell. Biol..

[2] Beck M., Förster F., Ecke M., Plitzko J.M., Melchior F., Gerisch G., Baumeister W., Medalia O. (2004). Nuclear Pore Complex Structure and Dynamics Revealed by Cryoelectron Tomography. Science.

[3] Beck M., Medalia O. (2008). Structural and Functional Insights into Nucleocytoplasmic Transport. Histol. Histopathol..

[4] Beck M., Lučić V., Förster F., Baumeister W., Medalia O. (2007). Snapshots of Nuclear Pore Complexes in Action Captured by Cryo-electron Tomography. Nature.

[5] Crisp M., Liu Q., Roux K., Rattner J.B., Shanahan C., Burke B., Stahl P.D., Hodzic D. (2006). Coupling of the Nucleus and Cyto-plasm: Role of the LINC Complex. J. Cell. Biol..

[6] Schulz I., Baumann O., Samereier M., Zoglmeier C., Gräf R. (2009). Dictyostelium Sun1 is a Dynamic Membrane Protein of both Nuclear Membranes and Required for Centrosomal Association with Clustered Centromeres. Eur. J. Cell Biol..

[7] Xiong H., Rivero F., Euteneuer U., Mondal S., Mana-Capelli S., Larochelle D., Vogel A., Gassen B., Noegel A.A. (2008). *Dictyostelium* Sun-1 Connects the Centrosome to Chromatin and Ensures Genome Stability. Traffic.

[8] Tikhonenko I., Magidson V., Gräf R., Khodjakov A., Koonce M.P. (2013). A Kinesin-Mediated Mechanism that Couples Centrosomes to Nuclei. Cell. Mol. Life Sci..

[9] Tikhonenko I., Nag D.K., Robinson D.N., Koonce M.P. (2009). Microtubule-Nucleus Interactions in *Dictyostelium discoideum* Mediated by Central Motor Kinesins. Eukaryot. Cell.

[10] Catalano A., O’Day D.H. (2011). Nucleolar Localization and Identification of Nuclear/Nucleolar Localization Signals of the Calmodulin-Binding Protein Nucleomorphin during Growth and Mitosis in *Dictyostelium*. Histochem. Cell. Biol..

[11] Krüger A., Batsios P., Baumann O., Luckert E., Schwarz H., Stick R., Meyer I., Gräf R. (2012). Characterization of Ne81, the First Lamin-Like Nucleoskeleton Protein in a Unicellular Organism. Mol. Biol. Cell.

[12] Mitic K., Meyer I., Gräf R., Grafe M. (2023). Temporal Changes in Nuclear Envelope Permeability during Semi-Closed Mitosis in *Dictyostelium* Amoebae. Cells.

[13] Kaller M., Euteneuer U., Nellen W. (2006). Differential Effects of Heterochromatin Protein 1 Isoforms on Mitotic Chromosome Dis-tribution and Growth in *Dictyostelium discoideum*. Eukaryot. Cell.

[14] Omura F., Fukui Y. (1985). Dictyostelium MTOC: Structure and Linkage to the Nucleus. Protoplasma.

[15] Gräf R., Grafe M., Meyer I., Mitic K., Pitzen V. (2021). The *Dictyostelium* Centrosome. Cells.

[16] Ueda M., Schliwa M., Euteneuer U. (1999). Unusual Centrosome Cycle in *Dictyostelium*: Correlation of Dynamic Behavior and Structural Changes. Mol. Biol. Cell.

[17] De Souza C.P., Osmani S.A. (2007). Mitosis, Not Just Open or Closed. Eukaryot. Cell.

[18] Mitic K., Grafe M., Batsios P., Meyer I. (2022). Partial Disassembly of the Nuclear Pore Complex Proteins during Semi-Closed Mitosis in *Dictyostelium discoideum*. Cells.

[19] Schweigel U., Batsios P., Müller-Taubenberger A., Gräf R., Grafe M. (2022). *Dictyostelium* Spastin is Involved in Nuclear Envelope Dynamics During Semi-closed Mitosis. Nucleus.

[20] Kessin R.H. (2001). Dictyostelium: Evolution, Cell Biology, and the Development of Multicellularity.

[21] Sameshima M. (1985). The Orientation of Nucleus, Nucleus-Associated Body and Protruding Nucleolus in Aggregating *Dictyostelium discoideum*. Exp. Cell. Res..

[22] Fawcett D.W. (1966). On the Occurrence of a Fibrous Lamina on the Inner Aspect of the Nuclear Envelope in Certain Cells of Vertebrates. Am. J. Anat..

[23] Gray E.G., Guillery R.W. (1963). An Electron Microscopical Study of the Ventral Nerve Cord of the Leech. Z. Zellforsch. Mikrosk. Anat. Vienna Austria.

[24] Beams H.W., Tahmisian T.N., Devine R., Anderson E. (1957). Ultrastructure of the Nuclear Membrane of a Gregarine Parasitic in Grasshoppers. Exp. Cell Res..

[25] Pappas G.D. (1956). The Fine Structure of the Nuclear Envelope of Amoeba Proteus. J. Biophys. Biochem. Cytol..

[26] Gerace L., Blum A., Blobel G. (1978). Immunocytochemical Localization of the Major Polypeptides of the Nuclear Pore Complex-Lamina Fraction. Interphase and Mitotic Distribution. J. Cell Biol..

[27] Gerace L., Blobel G. (1982). Nuclear Lamina and the Structural Organization of the Nuclear Envelope. Cold Spring Harb. Symp. Quant. Biol..

[28] Goldman A.E., Maul G., Steinert P.M., Yang H.Y., Goldman R.D. (1986). Keratin-like Proteins That Coisolate with Intermediate Filaments of BHK-21 Cells Are Nuclear Lamins. Proc. Natl. Acad. Sci. USA.

[29] Gruenbaum Y., Foisner R. (2015). Lamins: Nuclear Intermediate Filament Proteins with Fundamental Functions in Nuclear Mechanics and Genome Regulation. Annu. Rev. Biochem..

[30] Wong X., Melendez-Perez A.J., Reddy K.L. (2022). The Nuclear Lamina. Cold Spring Harb. Perspect. Biol..

[31] Adam S.A., Sengupta K., Goldman R.D. (2008). Regulation of Nuclear Lamin Polymerization by Importin Alpha. J. Biol. Chem..

[32] Xie W., Chojnowski A., Boudier T., Lim J.S., Ahmed S., Ser Z., Stewart C., Burke B. (2016). A-type Lamins Form Distinct Filamentous Networks with Differential Nuclear Pore Complex Associations. Curr. Biol..

[33] Shimi T., Kittisopikul M., Tran J., Goldman A.E., Adam S.A., Zheng Y., Jaqaman K., Goldman R.D. (2015). Structural organization of nuclear lamins A, C, B1, and B2 revealed by superresolution microscopy. Mol. Biol. Cell.

[34] Naetar N., Ferraioli S., Foisner R. (2017). Lamins in the Nuclear Interior − Life Outside the Lamina. J. Cell Sci..

[35] Schulz I., Erle A., Gräf R., Krüger A., Lohmeier H., Putzler S., Samereier M., Weidenthaler S. (2009). Identification and Cell Cycle-dependent Localization of Nine Novel, Genuine Centrosomal Components in *Dictyostelium discoideum*. Cell Motil. Cytoskelet..

[36] Koreny L., Field M.C. (2016). Ancient Eukaryotic Origin and Evolutionary Plasticity of Nuclear Lamina. Genome Biol. Evol..

[37] Grafe M., Batsios P., Meyer I., Lisin D., Baumann O., Goldberg M.W., Gräf R. (2019). Supramolecular Structures of the Dictyostelium Lamin NE81. Cells.

[38] Grafe M., Hofmann P., Batsios P., Meyer I., Gräf R. (2020). In Vivo Assembly of a *Dictyostelium* Lamin Mutant Induced by Light, Mechanical Stress, and pH. Cells.

[39] Kollmar M. (2015). Polyphyly of Nuclear Lamin Genes Indicates an Early Eukaryotic Origin of the Metazoan-type Intermediate Filament Proteins. Sci. Rep..

[40] Preisner H., Habicht J., Garg S.G., Gould S.B. (2018). Intermediate Filament Protein Evolution and Protists. Cytoskelet. Hoboken.

[41] Schirmer E.C., Guan T., Gerace L. (2001). Involvement of the Lamin Rod Domain in Heterotypic Lamin Interactions Important for Nuclear Organization. J. Cell Biol..

[42] Wilson K.L. (2000). The Nuclear Envelope, Muscular Dystrophy and Gene Expression. Trends Cell Biol..

[43] Prüfert K., Vogel A., Krohne G. (2004). The Lamin CxxM Motif Promotes Nuclear Membrane Growth. J. Cell Sci..

[44] Shimi T., Butin-Israeli V., Adam S.A., Goldman R.D. (2011). Nuclear Lamins in Cell Regulation and Disease. Cold Spring Harb. Symp. Quant. Biol..

[45] Batsios P., Ren X., Baumann O., Larochelle D.A., Gräf R. (2016). Src1 is a Protein of the Inner Nuclear Membrane Interacting with the *Dictyostelium* Lamin NE81. Cells.

[46] Stewart-Hutchinson P., Hale C.M., Wirtz D., Hodzic D. (2008). Structural Requirements for the Assembly of LINC Complexes and Their Function in Cellular Mechanical Stiffness. Exp. Cell Res..

[47] Kirby T.J., Lammerding J. (2018). Emerging Views of the Nucleus as a Cellular Mechanosensor. Nat. Cell Biol..

[48] Maurer M., Lammerding J. (2019). The Driving Force: Nuclear Mechanotransduction in Cellular Function, Fate, and Disease. Annu. Rev. Biomed. Eng..

[49] Jahed Z., Mofrad M.R. (2019). The Nucleus Feels the Force, LINCed in or Not!. Curr. Opin. Cell Biol..

[50] Iyer K.V., Taubenberger A., Zeidan S.A., Dye N.A., Eaton S., Jülicher F. (2021). Apico-Basal Cell Compression Regulates Lamin A/C Levels in Epithelial Tissues. Nat. Commun..

[51] Wallace M., Fedorchak G.R., Agrawal R., Gilbert R.M., Patel J., Park S., Paszek M., Lammerding J. (2023). The Lamin A/C Ig-fold Undergoes Cell Density-Dependent Changes That Alter Epitope Binding. Nucleus.

[52] Ihalainen T.O., Aires L., Herzog F.A., Schwartlander R., Moeller J., Vogel V. (2015). Differential Basal-to-apical Accessibility of Lamin A/C Epitopes in the Nuclear Lamina Regulated by Changes in Cytoskeletal Tension. Nat. Mater..

[53] Szczesny S.E., Mauck R.L. (2017). The Nuclear Option: Evidence Implicating the Cell Nucleus in Mechanotransduction. J. Biomech. Eng..

[54] Swift J., Ivanovska I.L., Buxboim A., Harada T., Dingal P.C.D.P., Pinter J., Pajerowski J.D., Spinler K.R., Shin J.-W., Tewari M. (2013). Nuclear Lamin-A Scales with Tissue Stiffness and Enhances Matrix-Directed Differentiation. Science.

[55] Buxboim A., Swift J., Irianto J., Spinler K.R., Dingal P.D.P., Athirasala A., Kao Y.-R.C., Cho S., Harada T., Shin J.-W. (2014). Matrix Elasticity Regulates Lamin-A,C Phosphorylation and Turnover with Feedback to Actomyosin. Curr. Biol..

[56] Kochin V., Shimi T., Torvaldson E., Adam S.A., Goldman A., Pack C.-G., Melo-Cardenas J., Imanishi S.Y., Goldman R.D., Eriksson J.E. (2014). Interphase Phosphorylation of Lamin A. J. Cell Sci..

[57] Hashimura H., Morimoto Y.V., Hirayama Y., Ueda M. (2022). Calcium Responses to External Mechanical Stimuli in the Multicellular Stage of *Dictyostelium discoideum*. Sci. Rep..

[58] Lombardi M., Knecht D., Lee J. (2008). Mechano-chemical signaling Maintains the Rapid Movement of *Dictyostelium* Cells. Exp. Cell Res..

[59] Srivastava N., Traynor D., Piel M., Kabla A.J., Kay R.R. (2020). Pressure Sensing through Piezo Channels Controls Whether Cells Migrate with Blebs or Pseudopods. Proc. Natl. Acad. Sci. USA.

[60] Turgay Y., Eibauer M., Goldman A.E.G.T.S.R.D., Shimi T., Khayat M., Ben-Harush K., Dubrovsky-Gaupp A., Sapra T., Goldman R.D., Medalia Y.O. (2017). The Molecular Architecture of Lamins in Somatic Cells. Nature.

[61] Odell J., Gräf R., Lammerding J. (2024). Heterologous Expression of *Dictyostelium discoideum* NE81 in Mouse Embryo Fibroblasts Reveals Conserved Mechanoprotective Roles of Lamins. Mol. Biol. Cell.

[62] Batsios P., Meyer I., Gräf R. (2016). Proximity-Dependent Biotin Identification (BioID) in *Dictyostelium* Amoebae. Methods Enzym..

[63] Hagan I., Yanagida M. (1995). The Product of the Spindle Formation Gene Sad1+ Associates with the Fission Yeast Spindle Pole Body and Is Essential for Viability. J. Cell. Biol..

[64] Malone C.J., Fixsen W.D., Horvitz H.R., Han M. (1999). UNC-84 Localizes to the Nuclear Envelope and Is Required for Nuclear Migration and Anchoring during *C. elegans* Development. Development.

[65] McGillivary R.M., Starr D.A., Luxton G.W.G. (2023). Building and Breaking Mechanical Bridges Between the Nucleus and Cytoskeleton: Regulation of LINC Complex Assembly and Disassembly. Curr. Opin. Cell Biol..

[66] Majumder S., Willey P.T., DeNies M.S., Liu A.P., Luxton G.W.G. (2018). A Synthetic Biology Platform for the Reconstitution and Mechanistic Dissection of LINC Complex Assembly. J. Cell Sci..

[67] Cruz V.E., Demircioglu F.E., Schwartz T.U. (2020). Structural Analysis of Different LINC Complexes Reveals Distinct Binding Modes. J. Mol. Biol..

[68] Majumder S., Hsu Y.-Y., Moghimianavval H., Andreas M., Giessen T.W., Luxton G.W.G., Liu A.P. (2022). In Vitro Synthesis and Reconstitution Using Mammalian Cell-Free Lysates Enables the Systematic Study of the Regulation of LINC Complex Assembly. Biochemistry.

[69] Belaadi N., Guilluy C. (2024). Life Outside the LINC Complex—Do SUN Proteins Have LINC-independent Functions?. BioEssays.

[70] Jahed Z., Domkam N., Ornowski J., Yerima G., Mofrad M.R.K. (2021). Molecular Models of LINC Complex Assembly at the Nuclear Envelope. J. Cell Sci..

[71] Gurusaran M., Davies O.R., Kingdom U. (2021). A Molecular Mechanism for LINC Complex Branching by Structurally Diverse SUN-KASH 6:6 Assemblies. eLife.

[72] Jahed Z., Fadavi D., Vu U.T., Asgari E., Luxton G.W.G., Mofrad M.R. (2018). Molecular Insights into the Mechanisms of SUN1 Oligomerization in the Nuclear Envelope. Biophys. J..

[73] Shimada N., Inouye K., Sawai S., Kawata T. (2011). SunB, a Novel Sad1 and UNC-84 Domain-Containing Protein Required for Development of *Dictyostelium discoideum*. Dev. Growth Differ..

[74] Ponte E., Rivero F., Fechheimer M., Noegel A., Bozzaro S. (2000). Severe Developmental Defects in Dictyostelium Null Mutants for Actin-binding Proteins. Mech. Dev..

[75] Luxton G.W.G., A Starr D. (2014). KASHing up with the Nucleus: Novel Functional Roles of KASH Proteins at the Cytoplasmic Surface of the Nucleus. Curr. Opin. Cell Biol..

[76] Meyerzon M., Fridolfsson H.N., Ly N., McNally F.J., Starr D.A. (2009). UNC-83 is a Nuclear-specific Cargo Adaptor for Kinesin-1-Mediated Nuclear Migration. Development.

[77] Roux K.J., Crisp M.L., Liu Q., Kim D., Kozlov S., Stewart C.L., Burke B. (2009). Nesprin 4 is an Outer Nuclear Membrane Protein That Can Induce Kinesin-mediated Cell Polarization. Proc. Natl. Acad. Sci. USA.

[78] Batsios P., Gräf R., Koonce M.P., Larochelle D.A., Meyer I. (2019). Nuclear Envelope Organization in *Dictyostelium discoideum*. Int. J. Dev. Biol..

[79] Malone C.J., Misner L., Le Bot N., Tsai M.-C., Campbell J.M., Ahringer J., White J.G. (2003). The C. elegans Hook Protein, ZYG-12, Mediates the Essential Attachment between the Centrosome and Nucleus. Cell.

[80] Rehberg M., Kleylein-Sohn J., Faix J., Ho T.H., Schulz I., Gräf R. (2005). Dictyostelium LIS1 Is a Centrosomal Protein Required for Microtubule/Cell Cortex Interactions, Nucleus/Centrosome Linkage, and Actin Dynamics. Mol. Biol. Cell..

[81] Meyer I., Kuhnert O., Gräf R. (2011). Functional Analyses of Lissencephaly-related Proteins in *Dictyostelium*. Semin. Cell Dev. Biol..

[82] Kuhnert O., Baumann O., Meyer I., Gräf R. (2012). Functional Characterization of CP148, a Novel Key Component for Centrosome Integrity in Dictyostelium. Cell. Mol. Life Sci..

[83] Mana-Capelli S., Gräf R., Larochelle D.A. (2009). *Dictyostelium discoideum* CenB Is a Bona Fide Centrin Essential for Nuclear Architecture and Centrosome Stability. Eukaryot. Cell.

[84] Blau-Wasser R., Euteneuer U., Xiong H., Gassen B., Schleicher M., Noegel A.A. (2009). CP250, a Novel Acidic Coiled-Coil Protein of the Dictyostelium Centrosome, Affects Growth, Chemotaxis, and the Nuclear Envelope. Mol. Biol. Cell.

[85] Prosser S.L., Pelletier L. (2017). Mitotic Spindle Assembly in Animal Cells: A Fine Balancing Act. Nat. Rev. Mol. Cell Biol..

[86] Krüger S., Pfaff N., Gräf R., Meyer I. (2024). Dynamic Mitotic Localization of the Centrosomal Kinases CDK1, Plk, AurK, and Nek2 in *Dictyostelium* Amoebae. Cells.

[87] King M.C., Drivas T.G., Blobel G. (2008). A Network of Nuclear Envelope Membrane Proteins Linking Centromeres to Microtubules. Cell.

[88] Fernández-Álvarez A., Bez C., O’Toole E.T., Morphew M., Cooper J.P. (2016). Mitotic Nuclear Envelope Breakdown and Spindle Nucleation Are Controlled by Interphase Contacts between Centromeres and the Nuclear Envelope. Dev. Cell.

[89] Schirmer E.C., Florens L., Guan T., Yates J.R., Gerace L. (2003). Nuclear Membrane Proteins with Potential Disease Links Found by Subtractive Proteomics. Science.

[90] Bone C.R., Tapley E.C., Gorjánácz M., Starr D.A. (2014). The *Caenorhabditis elegans* SUN Protein UNC-84 Interacts with Lamin to Transfer Forces from the Cytoplasm to the Nucleoskeleton during Nuclear Migration. Mol. Biol. Cell.

[91] Buch C., Lindberg R., Figueroa R., Gudise S., Onischenko E., Hallberg E. (2009). An Integral Protein of the Inner Nuclear Membrane Localizes to the Mitotic Spindle in Mammalian Cells. J. Cell Sci..

[92] Osorio D.S., Gomes E.R., Schirmer E.C., de las Heras J.I. (2014). Connecting the Nucleus to the Cytoskeleton for Nuclear Positioning and Cell Migration. Cancer Biology and the Nuclear Envelope.

[93] Bergqvist C., Niss F., Figueroa R.A., Beckman M., Maksel D., Jafferali M.H., Kulyté A., Ström A.-L., Hallberg E. (2019). Monitoring of Chromatin Organization in Live Cells by FRIC. Effects of the Inner Nuclear Membrane Protein Samp1. Nucleic Acids Res..

[94] Hiraoka Y., Maekawa H., Asakawa H., Chikashige Y., Kojidani T., Osakada H., Matsuda A., Haraguchi T. (2011). Inner Nuclear Membrane Protein Ima1 is Dispensable for Intranuclear Positioning of Centromeres. Genes Cells.

[95] Hou H., Zhou Z., Wang Y., Wang J., Kallgren S.P., Kurchuk T., Miller E.A., Chang F., Jia S. (2012). Csi1 Links Centromeres to the Nuclear Envelope for Centromere Clustering. J. Cell Biol..

[96] Barrales R.R., Forn M., Georgescu P.R., Sarkadi Z., Braun S. (2016). Control of Heterochromatin Localization and Silencing by the Nuclear Membrane Protein Lem2. Genes Dev..

[97] Brachner A., Foisner R. (2011). Evolvement of LEM Proteins as Chromatin Tethers at the Nuclear Periphery. Biochem. Soc. Trans..

[98] Gonzalez Y., Saito A., Sazer S. (2012). Fission Yeast Lem2 and Man1 Perform Fundamental Functions of the Animal Cell Nuclear Lamina. Nucleus.

[99] Laba J.K., Steen A., Popken P., Chernova A., Poolman B., Veenhoff L.M. (2015). Active Nuclear Import of Membrane Proteins Revisited. Cells.

[100] Capella M., Martín Caballero L., Pfander B., Braun S., Jentsch S. (2020). ESCRT Recruitment by the *S. cerevisiae* Inner Nuclear Membrane Protein Heh1 is Regulated by Hub1-mediated Alternative Splicing. J. Cell Sci..

[101] Stajdohar M., Rosengarten R.D., Kokosar J., Jeran L., Blenkus D., Shaulsky G., Zupan B. (2017). dictyExpress: A Web-based Platform for Sequence Data Management and Analytics in *Dictyostelium* and Beyond. BMC Bioinform..

[102] Mekhail K., Seebacher J., Gygi S.P., Moazed D. (2008). Role for Perinuclear Chromosome Tethering in Maintenance of Genome Stability. Nature.

[103] Yam C., Gu Y., Oliferenko S. (2013). Partitioning and Remodeling of the *Schizosaccharomyces japonicus* Mitotic Nucleus Require Chromosome Tethers. Curr. Biol..

[104] Liu H.-L., Osmani A.H., Osmani S.A. (2015). The Inner Nuclear Membrane Protein Src1 Is Required for Stable Post-Mitotic Progression into G1 in *Aspergillus nidulans*. PLoS ONE.

[105] Sameshima M., Fujimoto H., Imai Y., Tsukita S., Hashimoto Y. (1991). Relation of Nucleolar Structure and Position to the Cytoplasmic Microtubule System in *Dictyostelium*. Cell. Motil. Cytoskelet..

[106] Kono Y., Shimi T. (2024). Crosstalk between Mitotic Reassembly and Repair of the Nuclear Envelope. Nucleus.

[107] Sundquist W.I., Ullman K.S. (2015). Cell Biology. An ESCRT to Seal the Envelope. Science.

[108] Vietri M., Schink K.O., Campsteijn C., Wegner C.S., Schultz S.W., Christ L., Thoresen S.B., Brech A., Raiborg C., Stenmark H. (2015). Spastin and ESCRT-III Coordinate Mitotic Spindle Disassembly and Nuclear Envelope Sealing. Nature.

[109] Olmos Y., Perdrix-Rosell A., Carlton J.G. (2016). Membrane Binding by CHMP7 Coordinates ESCRT-III-Dependent Nuclear Envelope Reformation. Curr. Biol..

[110] Gu M., LaJoie D., Chen O.S., von Appen A., Ladinsky M.S., Redd M.J., Nikolova L., Bjorkman P.J., Sundquist W.I., Ullman K.S. (2017). LEM2 Recruits CHMP7 for ESCRT-mediated Nuclear Envelope Closure in Fission Yeast and Human Cells. Proc. Natl. Acad. Sci. USA.

[111] von Appen A., LaJoie D., Johnson I.E., Trnka M.J., Pick S.M., Burlingame A.L., Ullman K.S., Frost A. (2020). LEM2 Phase Separation Promotes ESCRT-mediated Nuclear Envelope Reformation. Nature.

[112] Lusk C.P., Ader N.R. (2020). CHMPions of Repair: Emerging Perspectives on Sensing and Repairing the Nuclear Envelope Barrier. Curr. Opin. Cell Biol..

[113] Bestul A.J., Yu Z., Unruh J.R., Jaspersen S.L. (2021). Redistribution of Centrosomal Proteins by Centromeres and Polo Kinase Controls Partial Nuclear Envelope Breakdown in Fission Yeast. Mol. Biol. Cell.

[114] Ader N.R., Chen L., Surovtsev I.V., Chadwick W.L., Rodriguez E.C., King M.C., Lusk C.P. (2023). An ESCRT Grommet Cooperates with a Diffusion Barrier to Maintain Nuclear Integrity. Nat. Cell Biol..

[115] Weijer C.J., Duschl G., David C.N. (1984). A Revision of the *Dictyostelium discoideum* Cell Cycle. J. Cell Sci..

[116] Hampoelz B., Andres-Pons A., Kastritis P., Beck M. (2019). Structure and Assembly of the Nuclear Pore Complex. Annu. Rev. Biophys..

[117] Kelley K., Knockenhauer K.E., Kabachinski G., Schwartz T.U. (2015). Atomic structure of the Y complex of the nuclear pore. Nat. Struct. Mol. Biol..

[118] Frey S., Rees R., Schünemann J., Ng S.C., Fünfgeld K., Huyton T., Görlich D. (2018). Surface Properties Determining Passage Rates of Proteins through Nuclear Pores. Cell.

[119] De Souza C.P., Osmani A.H., Hashmi S.B., Osmani S.A. (2004). Partial Nuclear Pore Complex Disassembly during Closed Mitosis in *Aspergillus nidulans*. Curr. Biol..

[120] Mcintosh J.R., Roos U.-P., Neighbors B., McDonald K.L. (1985). Architecture of the Microtubule Component of Mitotic Spindles from *Dictyostelium discoideum*. J. Cell Sci..

[121] Ding R., West R.R., Morphew D.M., Oakley B.R., McIntosh J.R. (1997). The Spindle Pole Body of *Schizosaccharomyces pombe* Enters and Leaves the Nuclear Envelope as the Cell Cycle Proceeds. Mol. Biol. Cell.

[122] Bouhlel I.B., Scheffler K., Tran P.T., Paoletti A. (2015). Monitoring SPB Biogenesis in Fission Yeast with High Resolution and Quantitative Fluorescent Microscopy. Methods Cell Biol..

[123] Uzawa S., Li F., Jin Y., McDonald K.L., Braunfeld M.B., Agard D.A., Cande W.Z. (2004). Spindle Pole Body Duplication in Fission Yeast Occurs at the G1/S Boundary but Maturation Is Blocked until Exit from S by an Event Downstream of *Cdc10*^+^. Mol. Biol. Cell.

[124] Bestul A.J., Yu Z., Unruh J.R., Jaspersen S.L. (2017). Molecular Model of Fission Yeast Centrosome Assembly Determined by Superresolution Imaging. J. Cell Biol..

[125] Dey G., Baum B. (2021). Nuclear Envelope Remodelling during Mitosis. Curr. Opin. Cell Biol..

[126] Dey G., Culley S., Curran S., Schmidt U., Henriques R., Kukulski W., Baum B. (2020). Closed Mitosis Requires Local Disassembly of the Nuclear Envelope. Nature.

[127] Tamm T., Grallert A., Grossman E.P., Alvarez-Tabares I., Stevens F.E., Hagan I.M. (2011). Brr6 Drives the *Schizosaccharomyces pombe* Spindle Pole Body Nuclear Envelope Insertion/Extrusion Cycle. J. Cell Biol..

[128] Lone M.A., Atkinson A.E., Hodge C.A., Cottier S., Martínez-Montañés F., Maithel S., Mène-Saffrané L., Cole C.N., Schneiter R. (2015). Yeast Integral Membrane Proteins Apq12, Brl1, and Brr6 Form a Complex Important for Regulation of Membrane Homeostasis and Nuclear Pore Complex Biogenesis. Eukaryot. Cell.

[129] Zhang W., Khan A., Vitale J., Neuner A., Rink K., Lüchtenborg C., Brügger B., Söllner T.H., Schiebel E. (2021). A Short Perinuclear Amphipathic α-helix in Apq12 Promotes Nuclear Pore Complex Biogenesis. Open Biol..

[130] Chen J., Gardner J.M., Yu Z., Smith S.E., McKinney S., Slaughter B.D., Unruh J.R., Jaspersen S.L. (2019). Yeast Centrosome Components form a Noncanonical LINC Complex at the Nuclear Envelope Insertion Site. J. Cell Biol..

[131] Rüthnick D., Schiebel E. (2016). Duplication of the Yeast Spindle Pole Body Once per Cell Cycle. Mol. Cell. Biol..

[132] Rüthnick D., Neuner A., Dietrich F., Kirrmaier D., Engel U., Knop M., Schiebel E. (2017). Characterization of Spindle Pole Body Duplication Reveals a Regulatory Role for Nuclear Pore Complexes. J. Cell Biol..

